# Children, adolescent, and youth mental health in Sri Lanka in the context of recent violence, COVID-19, and economic crisis: A call for action

**DOI:** 10.1016/j.lansea.2022.100021

**Published:** 2022-06-08

**Authors:** Sheikh Shoib, Miyuru Chandradasa, Layani Rathnayake, Sadia Usmani, Fahimeh Saeed

**Affiliations:** a Department of Psychiatry, Jawahar Lal Nehru Memorial Hospital, Srinagar, Kashmir, India; b Department of Psychiatry, University of Kelaniya, Ragama, Sri Lanka; c National Institute of Mental Health, Colombo, Sri Lanka; d Dow University of Health Sciences, Karachi, Pakistan; e Department of Psychiatry, Psychosis Research Center, University of Social Welfare and Rehabilitation Sciences, Tehran, Iran

Sri Lanka is a multi-religious South-Asian nation with 22 million people. Compared to its regional counterparts, Sri Lanka has a higher school life expectancy (14.11 years), better transition rates to secondary education (99.4%), and literacy rates for youth (99%), signifying the importance of schools in the well-being of children and adolescents.^1^

An armed conflict that lasted three decades, causing enormous physical and psychological trauma in North-eastern Sri Lanka, ended in 2009.^2^ A post-war era study in 2014 found a prevalence of emotional and behavioural problems at 13.8% in primary school children in Sri Lanka.^3^ The peace and stability over the following decade were disarranged by the Easter Sunday Bombings of Catholic Churches and luxury hotels in 2019, killing more than 250.^4^ In the following year, the world was ravaged by the COVID-19 pandemic, with more than 600,000 confirmed and probably many more unconfirmed cases in Sri Lanka. While COVID-19 is persisting, another fallout due to the depletion of foreign reserves occurred, creating a scarcity of imported fuel, gas, food items and medicine. School education was heavily affected by the lack of transportation for children and even the unavailability of paper to conduct examinations. Authorities have imposed rolling power cuts as a substantial proportion of the electricity production is dependent on imported fossil fuel, depriving school and university students of online learning time. This situation led to mass protests by youth in Colombo and the resignation of the cabinet en masse in April and the prime minister in May 2022.

The mental well-being of children, adolescents, and youth in Sri Lanka was affected due to Easter Sunday attack in 2019, COVID-19, and the foreign currency crisis in 2022. Disruption of schooling caused by the safety concerns of the Easter Sunday attacks further persisted during the pandemic. While online education offered a viable option in urban regions, large parts of Sri Lanka were not prepared due to the multifaceted digital divide.^5^ Further, staying home led to more engagement with electronic devices, and recent studies in Sri Lankan children have demonstrated an association between longer screen times with migraine, anxiety, poor sleep outcomes, and verbal and physical aggression.^6^^,^^7^

The current crisis has led to significant psychological distress and despair among children, adolescents, and youth. A survey among medical undergraduates during the COVID-19 pandemic found depressive symptoms in 40.8%, anxiety in 34.0%, and elevated stress levels in 24.7%.^8^ In a survey conducted in Eastern Sri Lanka, 75% of the school students studied had increased stress and anxiety due to COVID-19. The reasons were identified as closed schools, physical isolation, and unexpected changes in their lives.^9^ Further, increased digital screen time, mobile phone usage, disruption of learning activities, inability to engage in sports, and aesthetic activities may have contributed to the worsening mental well-being. Psychological distress is likely to worsen in the coming months with further disruption to the daily routine of school students, undergraduates, and their parents. Also, longitudinal studies have found that children born during the pandemic have lower verbal, motor, and cognitive skill acquirements than pre-pandemic born children, which is more in lower socioeconomic families.^10^

Children, adolescents, and youth in Sri Lanka have experienced significant psychosocial stressors, making them more vulnerable to developing psychological morbidity. However, the country's child and adolescent mental health services are primarily hospital-based, and there are less than ten child and adolescent psychiatrists practising in the country.^11^ Further, most Sri Lankan schools lack structured and standardised counselling services due to negative perceptions and unwillingness to invest in mental health.^12^ Therefore, immediate measures need to be taken to uplift the psychological well-being of this population, normalise their social environment, and recommendations are shown in Panel box 1.

**Panel box1:** Recommendation to improve the psychological well-being of children, adolescents, and youth in Sri Lanka.

Short-term Conduct an Island-wide survey on psychological morbidity through schools using a validated tool such as the Strength and Difficulties Questionnaire^13^^,^^14^ Empower the existing National Coordinating Committee on School Health by appointing mental health, child health, and educational professionals to implement measures to improve the emotional well-being Professional organisations related to child health request the government and parents to maintain schooling, ensure children's safety during protests and minimise exposure to violence Strengthen the capacity of the mental health helpline/chatline 1926 by the National Institute of Mental health to cater for children and adolescents and increase the awareness of this service through national media and social media platforms Allocate designated time and space for mental health promotional activities on government and private-owned electronic and printed media Monitor social and other media for the spread of false information and remove material promoting cruelty to prevent youth unrest and mob violence Create youth-friendly informative videos, posts, and online spaces on social media about psychological well-being, reconciliation, social responsibility, and leadership Provide easy access to mental health support through social media forums and instant messaging services in collaboration with the Ministry of Health Run mental health promotional activities for university students through departments of psychiatry and psychology already established in all state universities in Sri Lanka Long-term Provide more human resources such as medical officers, nursing officers, and allied health professionals and funding to government child and adolescent mental health services Prepare national screentime guidelines to guide parents and teachers on the healthy use of electronic devices in collaboration with the Sri Lanka College of Child and Adolescent Psychiatrists and other professional organisations

## Contributors

SS: conceptualisation, methodology, writing – review and editing. MC: writing – original draft, writing – review and editing. LR, SU and FS: writing – review and editing.

## Declaration of interests

Authors declare no competing interests.

## Appendix Supplementary materials

Image, application 1
