# Supplementary material for: Children, adolescent, and youth mental health in Sri Lanka in the context of recent violence, COVID-19, and economic crisis: A call for action
Source: Lancet Reg Health Southeast Asia. 2022 Jun 8;2:100021. doi: 10.1016/j.lansea.2022.100021 (PMC10306051; doi:10.1016/j.lansea.2022.100021)
Supplement: Supplementary file 1 [file mmc1.docx]

**Title Page**

Children, adolescent, and youth mental health in Sri Lanka in the context of Easter Sunday attack, COVID-19, and foreign currency crisis: a call for action

Sheikh Shoib^1^, Miyuru Chandradasa^2^, Layani Rathnayake^3^, Sadia Usmani^4^, Fahimeh Saeed^5^

1. Department of Psychiatry, Jawahar Lal Nehru Memorial Hospital, Srinagar, Kashmir, India.

Email: [Sheikhshoib22@gmail.com](mailto:Sheikhshoib22@gmail.com)

<https://orcid.org/0000-0002-3739-706X>

1. Department of Psychiatry, University of Kelaniya, Ragama, Sri Lanka

Email: [miyuruc@kln.ac.lk](mailto:miyuruc@kln.ac.lk)

<https://orcid.org/0000-0002-1873-8228>

1. National Institute of Mental Health, Colombo, Sri Lanka

Email: [layanirathnayake@gmail.com](mailto:layanirathnayake@gmail.com)

<https://orcid.org/0000-0003-0536-9272>

1. Dow University of Health Sciences, Karachi, Pakistan

Email: [dr.sadiausmani@gmail.com](mailto:dr.sadiausmani@gmail.com)

<https://orcid.org/0000-0003-2151-5874>

1. Department of Psychiatry, Psychosis Research Center, University of Social Welfare and Rehabilitation Sciences, Tehran, Iran

Email: [Fa.saeed@uswr.ac.ir](mailto:Fa.saeed@uswr.ac.ir)

<https://orcid.org/0000-0002-0214-3887>
